# Data-Fusion-Based Algorithm for Assessing Threat Levels of Low-Altitude and Slow-Speed Small Targets

**DOI:** 10.3390/s25175510

**Published:** 2025-09-04

**Authors:** Wei Wu, Wenjie Jie, Angang Luo, Xing Liu, Weili Luo

**Affiliations:** 1 School of Electronic Information Engineering, Xi’an Technological University, Xi’an 710021, China; wuwei@xatu.edu.cn (W.W.); jiewenjie@st.xatu.edu.cn (W.J.); lxing@xatu.edu.cn (X.L.); 2 School of Mechatronic Engineering, Xi’an Technological University, Xi’an 710021, China; 17639563013@163.com

**Keywords:** Low-Altitude Slow Small (LSS) Targets, threat assessment, data fusion, combined weighting method, D-S evidence theory

## Abstract

Low-Altitude and Slow-Speed Small (LSS) targets pose significant challenges to air defense systems due to their low detectability and complex maneuverability. To enhance defense capabilities against low-altitude targets and assist in formulating interception decisions, this study proposes a new threat assessment algorithm based on multisource data fusion under visible-light detection conditions. Firstly, threat assessment indicators and their membership functions are defined to characterize LSS targets, and a comprehensive evaluation system is established. To reduce the impact of uncertainties in weight allocation on the threat assessment results, a combined weighting method based on bias coefficients is proposed. The proposed weighting method integrates the analytic hierarchy process (AHP), entropy weighting, and CRITIC methods to optimize the fusion of subjective and objective weights. Subsequently, Technique for Order Preference by Similarity to an Ideal Solution (TOPSIS) and Dempster–Shafer (D-S) evidence theory are used to calculate and rank the target threat levels so as to reduce conflicts and uncertainties from heterogeneous data sources. Finally, the effectiveness and reliability of the two methods are verified through simulation experiments and measured data. The experimental results show that the TOPSIS method can significantly discriminate threat values, making it suitable for environments requiring rapid distinction between high- and low-threat targets. The D-S evidence theory, on the other hand, has strong anti-interference capability, making it suitable for environments requiring a balance between subjective and objective uncertainties. Both methods can improve the reliability of threat assessment in complex environments, providing valuable support for air defense command and control systems.

## 1. Introduction

Low-altitude airspace holds immense potential for military, agricultural, medical, and transportation applications [1,2]. Advancements in unmanned aerial vehicle technology have further highlighted its value [3,4]. While opening low-altitude airspace benefits economic development, global security conditions remain concerning or, in some cases, critical [5]. In recent years, rapid technological progress has diversified low-altitude airborne objects; however, illegal activities involving LSS targets have surged annually [6,7]. Rapid and accurate threat assessment of low-altitude targets can provide crucial auxiliary support for tasks such as air combat defense, firepower allocation, and strategy optimization. Monitoring LSS targets and providing early warnings have thus become an important research direction for modern defense systems.

LSS targets are defined as low-altitude objects flying below 1000 m at speeds under 200 km/h, with radar cross-sections (RCSs) smaller than 2 m^2^. Their covert operations and low detectability pose substantial threats to important protected target territories. Timely identification of incoming targets is vital for low-altitude air defense. In low-altitude airspace over urban areas and battlefields, accurate recognition and handling of LSS targets present major challenges for surveillance activities linked to defense operations and early warning systems [5,8,9]. Visible-light cameras are now a common means of LSS target identification and surveillance. Therefore, there is a growing need to build threat assessment algorithms for LSS targets based on visible camera detection techniques [10,11]. Currently, threat assessment relies on analyzing trajectories obtained through detection and tracking [12,13]. The general threat assessment process mainly consists of threat modeling for incoming targets, metric assignment using multi-attribute decision theory, and ranking, along with other activities [14]. The construction of a target threat assessment model usually involves two key aspects: first, reasonably selecting target threat assessment factors to form quantitative evaluation indicators; second, determining the weight of each evaluation factor [15].

The first key component can be distilled into effectively inferring target intent based on specific features extracted from images. The target type and its motion characteristics are the most intuitive, effective, and widely used attributes in threat assessment [16,17,18]. Threat level evaluation aims to quantitatively describe hostile intent [19]. Existing methods, such as multi-attribute decision-making (MADM) algorithms [20,21] and Bayesian networks [22,23], have made valuable contributions to threat identification strategies, but they also have certain limitations. The second key component, the weights of evaluation factors, can be determined using numerous methods. Depending on the data sources used for weight determination, these methods are categorized as subjective, objective, and combined weighting. Subjective weighting methods are among the earliest proposed and most maturely applied approaches. Techniques such as the Delphi Method, Best–Worst Method, AHP, and Analytic Network Process (ANP) have been widely adopted across various fields. Faizi et al. [24] refined the BWM by proposing a Linear Best–Worst Method and a Euclidean Best–Worst Method. Rehman et al. [25] constructed decision matrices based on the AHP with consistent fuzzy preference relations, both achieving enhanced expert decision quality in multi-criteria decision-making. Fei et al. [26] integrated D-number theory into the ANP to better accommodate uncertainty in expert judgments. Lin [27] introduced a Bayesian cosine maximization method to modify comparison matrices, enabling more accurate priority vector estimation. Despite challenges such as rank reversal, comparison scale inconsistency, and priority derivation limitations, the AHP remains the most efficient and practical decision-making method [28]. Luo et al. [29] introduced a target threat assessment model that combines the AHP and information entropy to determine the weights of targets’ subjective and objective threat factors.

Unlike conventional aerial targets, LSS targets pose detection challenges such as incomplete information acquisition [30]. In addition, some types of LSS targets also have flexible maneuverability and dynamic task planning capabilities, making it difficult to accurately assign weights to each threat factor using a single weighting method. Therefore, extracting complete, comprehensive feature information with strong discriminative ability from images and comprehensively utilizing these features to improve the performance of spatial target recognition is of great significance [31]. The subjective weighting approaches commonly used in target threat assessment require extensive auxiliary systems and prior knowledge bases. However, research on LSS targets is still in its infancy, and obtaining accurate assessment results based on the AHP method is difficult. The entropy method [32] and CRITIC method [11,13] can update weights when threat factors change, with high feasibility. This study attempts to introduce the AHP, entropy, and CRITIC methods into the threat assessment of LSS targets to establish an optimization model. The weights determined by the three methods are fused to obtain more reliable evaluation weights. Theoretically available multi-information fusion methods include the weighted average method [33], Kalman filtering method [34], Bayesian estimation method [22], D-S evidence theory [35], etc.

Prior research has proven that Bayesian estimation can be used to address information uncertainty [36]. However, the probability values calculated with this method are based on existing data, which is not applicable to the currently limited LSS target data. The TOPSIS (Technique for Order of Preference by Similarity to Ideal Solution) method is renowned for its effectiveness and robustness in managing attribute conflicts and generating exhaustive rankings. This is achieved through comparative analysis of alternatives against the positive ideal solution (PIS) and negative ideal solution (NIS) [37]. The key focus of the TOPSIS method is on weight determination. Weight assignments range from subjective (based on expert judgment) to objective (derived from data analysis). While subjective methods, such as Delphi and the analytic hierarchy process, can become complex as the number of criteria increases, objective strategies, such as Criteria Importance Through CRITIC and information entropy theory, provide statistical weights that reduce subjectivity [38]. D-S evidence theory is a typical mathematical tool for information fusion. It fuses uncertain information without prior knowledge or conditional probabilities and uses basic probability assignments to represent probabilities in uncertain problems [39]. D-S evidence theory is more advantageous when dealing with the diversity, incompleteness, inaccuracy, or uncertainty of knowledge and information in the military field [40]. Consequently, D-S evidence theory has been applied across intelligent decision-making domains, including surveillance systems [41], decision analysis [42], fault diagnosis [43], and target tracking [44]. Leveraging its unique strengths, D-S evidence theory reduces uncertainties during data fusion and holds significant potential in threat assessment [19]. The generalized D-S evidence theory has been proposed to address the issues of fusing potentially incomplete and conflicting information caused by an incomplete recognition framework, and it has been validated in practical fuzzy applications [45]. Therefore, this study considers modeling with the generalized D-S evidence theory to fuse incomplete information in LSS target threat assessment situations and compares the target threat evaluation results with those of multiple fusion algorithms to verify the effectiveness of this algorithm. The main contributions of this paper are as follows:

(1) A threat assessment system for LSS targets is established. Starting from aspects such as LSS target characteristics, flight attributes, and protected objects, an LSS threat index evaluation system is constructed by comprehensively considering target intentions and environmental attributes. Finally, the threat membership function is established for each attribute.

(2) By combining subjective and objective weighting methods with multi-attribute decision-making theory, the fuzziness inherent in visible-light camera data acquisition errors or subjective judgments is retained, and the dynamic characteristics of objective weights in target threat assessment are integrated. To effectively integrate information on the target’s combat intention, a combined weighting method based on deviation coefficients is proposed.

(3) Based on the weight values, the TOPSIS method and D-S evidence theory are used to rank the threats of different targets. The TOPSIS method comprehensively considers the membership degree of each attribute and uses a weighted scoring mechanism to improve the robustness of decision-making. D-S evidence theory is introduced to perform data fusion on the uncertain information of different LSS targets so as to rank the threat values of different targets. The effectiveness and accuracy of the TOPSIS method and D-S evidence theory are verified through testing and evaluation using different spatial LSS target data, and the two methods are compared with other fusion algorithms.

The structure of this article is as follows. In Section 2, we first describe the process of the LSS target threat assessment model and select the LSS target threat assessment factors and their membership functions. Section 3 elaborates on the models of the AHP, entropy weighting, CRITIC objective weighting, and combined weighting methods based on deviation coefficients. It also introduces the threat assessment system based on the TOPSIS method and D-S evidence theory. Section 4 verifies the method developed in this study through simulation and measured data and analyzes and discusses the experimental data. In Section 5, we summarize the research results and highlight future development directions.

## 2. LSS Target Threat Assessment Model and Quantification of Factors

### 2.1. Research on LSS Target Threat Assessment Methodology

Target threat assessment is a high-level information fusion process and generally involves three steps: (1) determining evaluation indicators; (2) formulating methods for calculating target threat levels; (3) ranking the calculated threat levels. Current research in the field of target threat assessment has relied on multiple theoretical frameworks, covering methods such as evidence theory, fuzzy reasoning, Bayesian networks, multi-attribute decision-making theory, cloud model theory, and neural networks. While these methods differ in their specific application objectives, their core principles generally make them extremely adaptable. When applied to LSS target threat assessment scenarios, each method, based on its unique theoretical basis, presents distinctive assessment characteristics. The application characteristics of each method are listed in Table 1.

In actual low-altitude defense environments, accurate assessment of target threat levels requires integrating multiple factors for comprehensive analysis and judgment. Although algorithms have significant advantages in rapid data processing, commanders’ experience and judgment still play an irreplaceable and crucial role in dynamic decision-making environments. Therefore, an algorithm’s capabilities must be synergistically integrated with the domain knowledge and professional experience of military professionals. When dealing with multi-target attributes and highly dynamic environments, multi-attribute decision-making theory has superior advantages in human–machine collaboration compared to other threat assessment methods.

**Figure 1:**
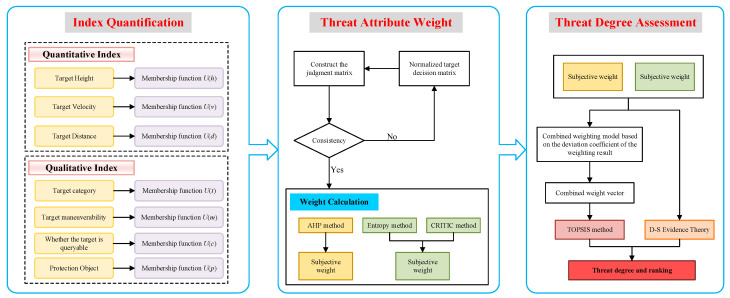
Flowchart of target threat assessment model.

LSS target recognition is also a key focus of target detection. Target recognition mainly achieves identification by collecting LSS images through imaging technologies, conducting comparative analysis, and extracting features. The maximum recognition distance based on visible-light imaging is approximately 1 km [50]. When conducting threat assessment for LSS targets detected by visible-light cameras, regarding each incoming target as a decision object transforms an aerial target threat assessment into an MADM problem. Decision criteria are grounded in the level of threat posed by targets to ground-based air defense systems. From the basic knowledge of multi-attribute decision theory, a threat assessment model is established, as shown in Figure 1. This threat assessment model first preprocesses target situational data and establishes different affiliation functions. Subsequently, the AHP, entropy weighting, and CRITIC methods are applied to calculate weight vectors. Spearman’s rank correlation coefficient is then utilized to compute combined weights. Finally, using TOPSIS and D-S evidence theory, the threat level for the target is comprehensively evaluated.

### 2.2. Selection and Quantification of Indicators for LSS Target Threat Assessment

To promote the development of low-altitude airspace resources and standardize the operation and management of these airspaces within urban areas, several laws, regulations, and key technical indicators applicable to LSS targets in urban areas have been implemented [51]. This provides an important reference for assessing the threat level of LSS targets. The threat assessment indicators for LSS targets are selected and quantified based on current detection technologies and their ability to reflect threat levels. Based on the nature and rationality of their membership functions, these indicators are divided into two categories: objective membership functions and subjective membership functions.

#### 2.2.1. Objective Membership Functions

Both the forms and parameters of objective membership functions are derived from physical principles, mathematical relationships, or statistical analysis of sensor data. They describe a clear and interpretable functional relationship between measurable physical quantities and the perceived threat levels.

(1) Target height

The target’s flight altitude impacts the success rate of surprise defense. The lower the altitude, the easier it is for targets to evade detection and defense. At the same time, low-altitude targets can reach our position faster. Conversely, targets at excessively high altitudes struggle to directly execute planned missions—i.e., the threat level increases as the target altitude decreases. Currently, the flight airspace for LSS targets worldwide is generally restricted to 60–300 m [51]. Based on an extensive data review, the detection capabilities of three mainstream detection methods (radar, infrared, and visible-light cameras) for LSS targets [29,30,50] are listed in Table 2.

From the above analysis, it can be seen that the effective detection range and recognition rate of visible-light cameras for ultra-low-altitude targets in complex urban backgrounds decrease significantly. When the flight altitude is 50 m, which is close to or lower than the height of typical urban high-rise building groups, targets can easily use terrain masking, increasing the difficulty of detection and interception. This is the critical threshold height for air defense systems. Therefore, this study sets the threat level to 1 when the altitude is below 50 m and uses a piecewise function as the height membership function *U*(*h*), where *h* in Equation (1) is the height of the LSS target:

(1) $$U\left(h\right)=\left\{\begin{array}{cc} 1 & h\leq 50\\ \frac{1}{1+{\left(h-50\right)}^{2}\times 10^{-3}} & h>50 \end{array}\right.$$

The quadratic function form is chosen to reflect the nonlinear accelerated growth in the threat level as altitude decreases, especially when approaching the 50 m critical height, where the threat increases sharply. The coefficient 10^−3^ is used to adjust the value range of the function within the domain h ∈ [50, 1000], ensuring that it falls within the interval [0, 1].

(2) Target velocity

The target’s flight velocity vector *U*(*v*) is related to both the speed magnitude v and the velocity direction. First, the target’s relative entry angle *θ* is calculated using Equation (2):

(2) θ=180+α−β

In the equation, *α* represents the target’s heading angle—the angle between its flight direction and north; *β* represents the target’s azimuth angle—the angle between the line connecting the target to the air defense center and north.

A higher target speed implies stronger maneuverability and a shorter flight time to reach our defense position, thus increasing the threat level. After determining the LSS target’s relative entry angle, its velocity component relative to the air defense center is calculated. The flight speed ranges of various LSS targets in low-altitude airspace are listed in Table 3. The median value of the speed range is selected as a reference, with the flight speed of LSS targets set to 40 m/s as the benchmark value. The membership function *U*(*v*) is shown in Equation (3):

(3) $$U\left(v\right)=\frac{\cos\left(\theta \right)\times v}{40}$$

In the equation, *v* denotes the speed of the LSS target, and *θ* denotes its relative approach angle.

(3) Target distance

The closer the target is to our position, the easier it is to carry out direct action and the shorter the response time for the defense system. Smaller detected target distance indicators imply a higher likelihood of endangering air defense positions and a greater threat posed by the target. With reference to the analysis of target detection capabilities in Table 2, the minimum distance of the target is set to 100 m [29]. This value has practical significance in low-altitude defense scenarios, usually indicating that the target has entered the effective kill zone of short-range defense systems or poses a direct collision threat. This distance indicator should meet the response time requirements of the defense system. The affiliation function for distance *U*(*d*) is expressed by Equation (4):

(4) $$U\left(d\right)=\left\{\begin{array}{cc} 1 & d\leq 100\\ \frac{1}{1+{\left(d-100\right)}^{2}\times 10^{-4}} & d>100 \end{array}\right.$$

The distance threat membership function *U*(*d*) adopts a quadratic form to simulate the nonlinear accelerated increase in the threat level as the distance decreases. The coefficient 10^−4^ ensures that the function values fall within the interval [0, 1] within the domain d ∈ [100, +∞).

#### 2.2.2. Subjective Membership Functions

Factors such as target type, maneuverability, query availability, and protected objects are subjective factors. The forms and parameters of these functions are mainly derived from the knowledge and tactical experience of domain experts. To enhance objectivity and consensus, this study invited 10 experts in related fields—3 personnel from air defense forces, 3 professors in the field of target detection systems, 3 UAV technicians, and 1 professor of military operations research. In the form of questionnaires, experts were asked to independently score and rank the threat levels of various targets, assign threat values corresponding to strong, medium, and weak maneuverability, identify threat differences between queryable and non-queryable targets, and provide relative threat values of typical protected objects. Scores were on a 0–1 scale, with 1 representing the maximum threat, and results were retained to one significant figure. The mean and median of each score were calculated, and the obtained values were compared with the research findings in references [29,51]. Finally, the membership values for factors such as target type, maneuverability, query availability, and protected objects were determined.

(1) Target type

**Figure 2:**
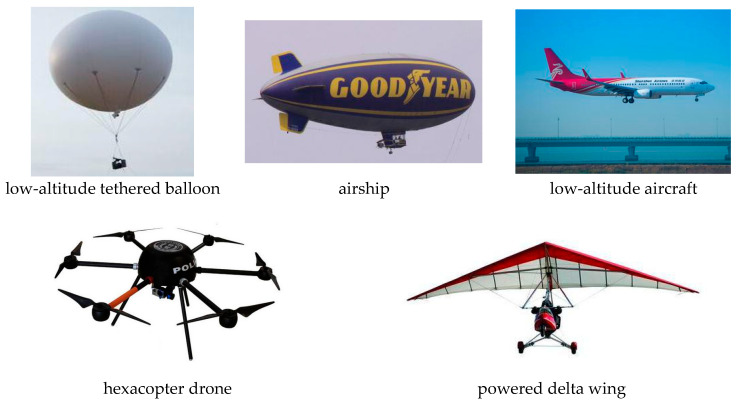
LSS target types.

In recent years, various types of aircraft have emerged in the market, such as paragliders, light helicopters, and gyroplanes. The LSS targets in this study are grouped into five categories: powered delta wings, hexacopter unmanned aerial vehicles (UAVs), airships, low-altitude airplanes, and low-altitude tethered balloons. Their classification is shown in Figure 2.

Based on expert opinions [51], the above LSS targets are ranked by threat level from highest to lowest as follows: hexacopter UAVs, powered delta wings, airships, low-altitude aerial photography balloons, and low-altitude airplanes. The affiliation function value *U*(*t*) is shown in Equation (5):

(5) $$U\left(t\right)=\left\{\begin{array}{ll} 1 & \mathrm{Hexacopter}\;\mathrm{drone}\\ 0.8 & \mathrm{Powered}\;\mathrm{delta}\;\mathrm{wing}\;\mathrm{aircraft}\\ 0.6 & \mathrm{Airship}\\ 0.4 & \mathrm{Low}-\mathrm{altitude}\;\mathrm{aerial}\;\mathrm{photography}\;\mathrm{of}\;\mathrm{balloons}\\ 0.1 & \mathrm{Low}\;\mathrm{altitude}\;\mathrm{aircraft} \end{array}\right.$$

(2) Target’s mobility performance

Maneuverability serves as a static indicator of target characteristics. Once the target type is identified, its corresponding maneuverability can be obtained. For air defense systems, targets with poorer maneuverability are easier to intercept. The mobility levels of the LSS target types under study are set to be strong, medium, and weak, and the affiliation function values *U*(*m*) corresponding to these three levels are shown in Equation (6):

(6) $$U\left(m\right)=\left\{\begin{array}{ll} 0.8 & \mathrm{Powerful}\\ 0.6 & \mathrm{Middle}\\ 0.1 & \mathrm{Weak} \end{array}\right.$$

(3) Whether the target is searchable

When targets can respond to ground communication signals, their threat level decreases. When targets fail to respond and are unqueryable, the threat level increases. Their affiliation function values *U*(*c*) are shown in Equation (7):

(7) $$U\left(c\right)=\left\{\begin{array}{ll} 0.1 & \mathrm{Searchable}\\ 0.3 & \mathrm{Failed}\;\mathrm{to}\;\mathrm{query} \end{array}\right.$$

(4) Protected object

The threat that an incoming LSS target poses to a protected site varies depending on the type of protected site. Here, the threat degree is set to 0.8, 0.6, or 0.5, and the specific discrete affiliation function values *U*(*p*) are shown in Equation (8):

(8) $$U\left(p\right)=\left\{\begin{array}{ll} 0.8 & \mathrm{Command}\;\mathrm{post}\\ 0.6 & \mathrm{Government}\;\mathrm{sector}\\ 0.6 & \mathrm{Important}\;\mathrm{event}\;\mathrm{venues}\\ 0.5 & \mathrm{City}\;\mathrm{center}\\ 0.5 & \mathrm{Vital}\;\mathrm{communication}\;\mathrm{line} \end{array}\right.$$

## 3. LSS Target Threat Assessment Model

The aim of the threat assessment model for LSS targets is to scientifically and reliably quantify the threat levels of targets; its core consists of two parts: weighted optimization of threat factors and fusion-based assessment of target threat levels. The process of assigning weights focuses on determining the relative importance of each threat assessment index. By integrating the advantages of subjective and objective weighting methods, their respective limitations can be overcome, and more reliable combined weights can be obtained. Based on the obtained comprehensive weights, multi-attribute decision-making and information fusion techniques can be used to comprehensively calculate and rank the overall threat level of a target, effectively handle the uncertain and conflicting data in the assessment process, and finally output reliable results of target threat level assessment.

### 3.1. Methods of Improvement

The core challenges in weight allocation for evaluating LSS target threat indicators are mainly as follows: the lack of sufficient historical data, leading to incomplete information; high uncertainty caused by the complex kinematic characteristics of target types and their vulnerability to countermeasures; and the need to integrate dynamic real-time data with static expert knowledge. A single weight-setting method cannot fully encompass such complexity. Therefore, a hybrid weighting scheme is proposed, which integrates three complementary methods, each addressing specific aspects of the LSS target problem.

The analytic hierarchy process (AHP) can tackle issues of incomplete information and a lack of statistical data. This method incorporates domain experts’ knowledge and experience, which is crucial for quantifying intangible attributes and providing an a priori understanding of threat dynamics. A consistency construction approach is adopted to meet the needs of rapid assessment in low-level security defense scenarios.

The entropy weight method is suitable for processing dynamic real-time data. It can allocate weights based on the degree of data dispersion among different targets. Indicators with high variability contribute more to distinguishing threat levels among current LSS targets, enabling the assessment to adapt to immediate situations.

The CRITIC method can reflect the relationships between indicators. It considers both the comparative intensity between indicators and their conflict characteristics, providing a more comprehensive objective measurement standard, which is vital for LSS target evaluation.

The aim of integrating the above three methods is to establish a robust weight foundation that is supported by professional knowledge, is responsive to data, and accounts for interactions between different criteria. This is essential for an environment full of uncertainties and evolving low-frequency sudden threats.

#### 3.1.1. Consistency-Based Analytic Hierarchy Process (AHP) Subjective Weights

**Figure 3:**
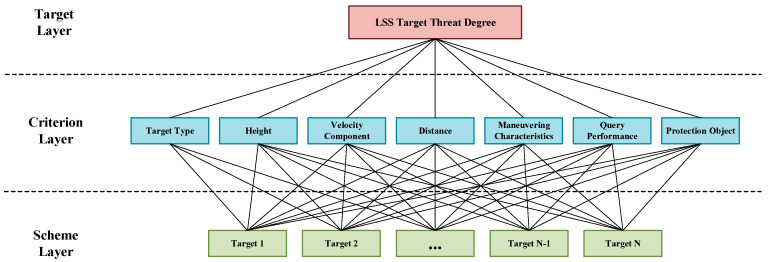
Hierarchical analysis model diagram.

The AHP is a multi-objective decision analysis method integrating qualitative and quantitative approaches. It uses expert experience to compare the importance of participating factors, constructs a comparison matrix to determine the relative importance of each factor in the hierarchy, and forms a judgment matrix to quantitatively determine the weights of threat factors [52]. In the threat assessment model, the AHP is converted into a problem of finding the threat degree of the attacking target; in this version, the target layer is the threat degree for the current attacking LSS target, the criterion layer comprises the threat attributes for the target type, altitude, and speed, and the program layer is the current attacking batch LSS target. The hierarchical model is shown in Figure 3.

The following steps are typically used when determining indicator weights using this method [53]:

**Step 1**: Forming a judgment matrix. To establish an AHP model for the threat assessment system, experts, based on experience, rank each target threat attribute to characterize its degree of importance. Values from 1 to 9 quantitatively represent the importance level for each threat element, as shown in Table 4. The judgment matrix *A* = (*A_ij_*)*_n×n_*, *a_ij_*, is formed to compare targets based on various threat attributes.

**Step 2**: Hierarchical ordering. Using matrix theory, the equation *Aw* = *λ*_max_*w* is solved to obtain the eigenvector *w* and the maximum eigenvalue *λ*_max_. The threat level weight vector for the indicator system is *w* = (*w*_1_, *w*_2_, …, *w_n_*)*^T^*.

**Step 3**: Conducting consistency tests. Expert evaluations are mainly subjective and do not follow mathematical formulas, making it difficult to meet the consistency requirements. Therefore, a consistency check of the calculated threat weight vector *w* is necessary.

**Step 4**: Hierarchical total ordering. When dealing with multiple hierarchical structures, if the weight vector of indices at the (*k* − 1)-th level is 1 and the weight vector of the *k*-th level relative to the (*k* − 1)-th level is *H*, then the weight vector of the *k*-th level relative to the target layer is *w* = (*w*_1_, *w*_2_, …, *w_n_*)*^T^* = *l* · *H*.

Since the consistency test increases modeling complexity, it is improved, and the steps of the improved AHP method are as follows:

**Step 1**: Based on the 1–9 ranking scale shown in Table 1, a judgment matrix *q* is generated through pairwise comparison of each target threat attribute element in the evaluation index set, as shown in Equation (9). When constructing the first row, the 1–9 scale is used as the criterion; for other rows, judgments are formed using *q_ij_* = *q_ik_* × *q_kj_*, and the complete judgment matrix *q* is finally established.

(9) $$q=\left[\begin{array}{cccc} q_{11} & q_{12} & \ldots & q_{1n}\\ q_{21} & q_{22} & \ldots & q_{2n}\\ \vdots & \vdots & \ddots & \vdots \\ q_{n1} & q_{n2} & \ldots & q_{nn} \end{array}\right]$$

**Step 2**: Using matrix theory, the equation *qw* = *λ*_max_*w* is solved to obtain the eigenvector *w* and the maximum eigenvalue *λ*_max_ of matrix *q*. The resulting vector *w* = (*w*_1_, *w*_2_, …, *w_n_*)*^T^* represents the threat weight vector of the indicator system.

#### 3.1.2. Entropy Method for Determining Objective Weights

After determining subjective weights based on expert experience, information entropy theory is introduced into the process of determining target attribute weights for LSS target threat assessment. This approach makes weight determination more objective and better reflects the impact of each attribute on threat assessment. With the entropy weight method, weights are assigned by analyzing the deviation of information entropy values, thereby deriving indicator weights with correct and reliable results. The entropy weight method is defined as follows.

If an object has *n* states, and the probability of each state is (*p*_1_, *p*_2_, …, *p_n_*), then the information entropy value of the object is defined as

(10) $$E\left(X\right)=E\left[\log\frac{1}{P\left(a_{i}\right)}\right]=-\sum_{i=1}^{q}P\left(a_{i}\right)\log P\left(a_{i}\right)$$

The steps for determining objective weights using the entropy method are as follows:

**Step 1**: Calculate the entropy value for the indicator *E_j_*. Construct a decision matrix *B* = (*b_ij_*)*_m × n_* based on threat assessment indicators, where *m* represents the number of LSS targets and *n* represents the number of threat attributes. Here, *b_ij_* denotes the attribute value of the *i*-th target under the *j*-th threat indicator. Normalize the decision matrix *B* to obtain the normalized matrix $\tilde{B}={\left({\tilde{b}}_{ij}\right)}_{m\times n}$, in which

(11) $${\tilde{b}}_{ij}=b_{ij}/\sum_{i=1}^{m}b_{ij}$$

where ${\tilde{b}}_{ij}$ is a value normalized to the attribute.

Then, the information entropy *E_j_* for the *j*-th threat assessment indicator is

(12) $$E_{j}=-\frac{1}{\ln m}\sum_{i=1}^{m}{\tilde{b}}_{ij}\ln{\tilde{b}}_{ij}$$

**Step 2**: Calculate the weighting system for each indicator. Based on the principles of the entropy weight method, the objective weight *w_oj_* for the *j*-th threat assessment indicator can be obtained as

(13) $$w_{oj}=\left(1-E_{j}\right)/\sum_{j=1}^{n}\left(1-E_{j}\right)$$

Then, the information entropy-based target weight value *w_o_* is

(14) $$w_{o}=\left[w_{o1},w_{o2},w_{o3},w_{o4},w_{o5},w_{o6},w_{o7}\right]$$

In this formula, *w_o_*_1_, *w_o_*_2_, *w_o_*_3_, *w_o_*_4_, *w_o_*_5_, *w_o_*_6_, and *w_o_*_7_ represent the calculated weights for the following threat factors: target category, target altitude, velocity component relative to the air defense center, target distance, target armament performance, whether the target is queryable, and the protected object, respectively.

#### 3.1.3. CRITIC Calculation of Objectively Assigned Weights

The CRITIC method, proposed by DIAKOULAKI, is an objective weighting method that assigns weights by quantifying the information volume of each indicator based on two key principles: comparative intensity (variability) and conflict (correlation) among indicators. Below are the specific calculation steps and an example application in threat assessment.

**Step 1**: The original situational data matrix *d_ij_*, formed by *j* threat assessment indicators of *i* targets, first undergoes normalization processing.

For positive indicators (the higher the value, the higher the threat, e.g., speed),

(15) $$d_{ij}^{*}=\frac{d_{ij}-\min\left(d_{j}\right)}{\max\left(d_{j}\right)-\min\left(d_{j}\right)}$$

For negative indicators (the smaller the value, the higher the threat, e.g., distance),

(16) $$d_{ij}^{*}=\frac{\max\left(d_{j}\right)-d_{ij}}{\max\left(d_{j}\right)-\min\left(d_{j}\right)}$$

**Step 2**: The comparative strength for the indicator *S_j_* is determined:

(17) $$\left\{\begin{array}{l} {\bar{d}}_{j}=\frac{1}{n}\sum_{i=1}^{n}d_{ij}\\ S_{j}=\sqrt{\frac{1}{m}\sum_{i=1}^{m}{\left(d_{ij}-{\bar{d}}_{j}\right)}^{2}} \end{array}\right.\begin{array}{cc} & j=1,2,\ldots ,n \end{array}$$

The conflictual correlation coefficient *r_ij_* is

(18) $$r_{ij}=\mathrm{cov}\left(D_{i},D_{j}\right)/\left(S_{i},S_{j}\right)\begin{array}{cc} & i,j=1,2,\ldots ,n \end{array}$$

**Step 3**: Conflictiveness is measured by the correlation coefficient between indicators: the higher the conflictiveness (the lower the correlation coefficient), the greater the weight. The formula for calculating conflictability is

(19) $$\mathrm{Conflict}=\sum_{j=1}^{n}\left(1-r_{ij}\right)$$

**Step 4**: The comprehensive information volume *G_j_* is determined for each threat indicator:

(20) $$G_{j}=S_{j}\sum_{i=1}^{n}\left(1-r_{ij}\right)\begin{array}{cc} & j=1,2,\ldots ,n \end{array}$$

**Step 5**: The weight coefficients, denoted by *w*, for each threat indicator are determined from the combined information of the indicators:

(21) $$\left\{\begin{array}{l} w=\left(w_{1},w_{2},\ldots ,w_{n}\right)\\ w_{j}=\frac{G_{j}}{\sum_{j=1}^{n}G_{j}} \end{array}\right.\begin{array}{cc} & j=1,2,\ldots ,n \end{array}$$

#### 3.1.4. Combined Assignment Method Based on Deviation Coefficients

In the threat assessment of LSS targets, the results of subjective and objective weighting methods often differ, reflecting uncertainties from different perspectives. Simple fixed-proportion fusion (such as equal-weight averaging) cannot fully utilize the consistency information between the results of these methods. The ideal fusion assigns greater weights to methods with more consistent results. To this end, this study proposes a dynamic fusion mechanism based on the weight deviation similarity index *r_lk_*. Methods with highly consistent weighting results can more truly reflect the weight relationships and should dominate the fusion; methods with large result differences should contribute less. Compared with fixed fusion or complex optimization methods, this consistency-based dynamic weighting strategy provides a more intuitive, transparent, and efficient approach to handling uncertainties in weight allocation, enhancing the robustness of fusion results.

Due to the limitations of uncertainties in various evaluation indicators and data collection conditions, existing evaluation schemes often only consider expert experience or original situational data. To make up for the drawbacks of subjective and objective weighting, this study, on the basis of the obtained subjective and objective weight vectors, determines their weighting coefficients in the weight combination based on the deviation coefficients from the subjective and objective weighting results mentioned above; then, the relevant models are established, and the combined weights are calculated.

The numerical deviation representing the differences between the results of different weighting methods is called the weight deviation similarity index *r_lk_*. The smaller the difference, the greater the consistency between the weighting methods, and the larger the *r_lk_* value. The value of *r_lk_* is always less than or equal to 1 and non-negative. The consistency coefficient *r_lk_* of the weighting results is defined as the deviation correlation coefficient between the *l*-th and *k*-th weighting methods:

(22) $$r_{lk}=1-\frac{\sum_{i=1}^{n}\left|w_{li}-w_{ki}\right|}{n}$$

The specific solution steps for model construction are as follows.

Let the indicator number for the original evaluation program be *i*, *i* = 1, 2, …, n, the assignment method used be *j*, *j* = 1, 2, …, n, and the weight of each indicator for each assignment method be *w* = (*w*_1_, *w*_2_, …, *w_n_*).

**Step 1**: By minimizing the sum of squared deviations, solve for the optimal combined weights. The calculation model is

(23) $$\left\{\begin{array}{l} \min\sum_{j=1}^{q}\sum_{i=1}^{n}l_{j}{\left(w_{i}-w_{ij}\right)}^{2}\\ st.\sum_{i=1}^{n}w_{i}=1,\begin{array}{cc} & w\geq 0 \end{array} \end{array}\right.$$

Formulate it as the Lagrangian function *L*(*l*, *w_i_*):

(24) $$L\left(l,w_{i}\right)=\sum_{j=1}^{q}\sum_{i=1}^{n}l_{i}{\left(w_{i}-w_{ij}\right)}^{2}+l\left(\sum_{i=1}^{n}w_{i}-1\right)=0$$

This leads to the unique optimal solution *w_i_*:

(25) $$w_{i}=\sum_{i=1}^{q}l_{j}w_{ij}\begin{array}{ccc} & & i=1,2,\ldots ,n \end{array}$$

**Step 2**: Using the concept of correlation coefficients, establish a relevant model to calculate the weight coefficients for the three weighting results. Let *r_lk_* denote the correlation coefficient between the *l*-th and *k*-th weighting methods, and obtain the correlation coefficient matrix *r* corresponding to *q* weighting results:

(26) $$r=\left[\begin{array}{cccc} r_{11} & r_{12} & \ldots & r_{1q}\\ r_{21} & r_{22} & \ldots & r_{2q}\\ \vdots & \vdots & \ddots & \vdots \\ r_{q1} & r_{q2} & \ldots & r_{qq} \end{array}\right]$$

From the definition of *r_ik_*, it is known that *r_ll_* = 1 (*l* = 1, 2, …, *q*), *r_lk_* = *r_kl_* (*l*, *k* = 1, 2, …, *q*). According to the symmetric matrix, calculate the correlation degree *r_j_* between the *j*-th weighting method and all other weighting methods. The calculation formula is

(27) $$r_{j}=\frac{1}{q-1}\sum_{k=1,k\neq l}^{q}r_{jk}$$

Through normalization, obtain the weight coefficients *l_j_* for each weighting method in the combined weighting:

(28) $$l_{j}=\frac{r_{j}}{\sum_{j=1}^{q}r_{j}}$$

Substitute the above formula into Equation (25) to obtain the combined weights *v* of each indicator calculated based on three weighting methods:

(29) $$v=\sum_{j=1}^{q}\frac{r_{j}}{\sum_{j=1}^{q}r_{j}}w_{ij},\begin{array}{ccc} & & i=1,2,\ldots ,n \end{array}$$

### 3.2. Target Threat Level Assessment

#### 3.2.1. Target Threat Level Assessment Based on TOPSIS Method

The TOPSIS method is a commonly used multi-attribute decision analysis approach. Its basic principle involves ranking alternatives by calculating their distances to both the ideal solution (the best possible scenario, where all attributes achieve optimal values) and the negative ideal solution (the worst possible scenario, where all attributes achieve suboptimal values). A better alternative is simultaneously closer to the positive ideal solution and farther from the negative ideal solution. After obtaining the combined weights v for target threat indicators, as described in the preceding section, we analyze and rank the threat levels of each target using the decision matrix and weight information.

The standardized attribute matrix *H* = [*h_ij_*]*_m×n_* is constructed using the comprehensive weights *v* = [*v*_1_, *v*_2_, …, *v_n_*] for target attributes, calculated using the combined weighting method based on deviation coefficients and based on the threat indicators of each target.

**Step 1**: Calculate the weighted normalized matrix *V*:

(30) $$V={\left[v_{ij}\right]}_{m\times n}={\left[v_{j}h_{ij}\right]}_{m\times n}$$

**Step 2**: Determine the ideal solution *v_j_*^+^ and negative ideal solution *v_j_*^−^:

(31) $$\left\{\begin{array}{l} v_{j}^{+}=\underset{1\leq i\leq n}{\max}\left(v_{ij}\right)\\ v_{j}^{-}=\underset{1\leq i\leq n}{\min}\left(v_{ij}\right) \end{array}\right.$$

**Step 3**: Calculate the distance *d_i_*^+^, *d_i_*^−^ from each target threat indicator attribute value series to the ideal and negative ideal solutions:

(32) $$\left\{\begin{array}{l} d_{i}^{+}=\sum_{j=1}^{m}\left\|v_{ij}-v_{j}^{+}\right\|\\ d_{i}^{-}=\sum_{j=1}^{m}\left\|v_{ij}-v_{j}^{-}\right\| \end{array}\right.$$

**Step 4**: Calculate the closeness coefficient *C_i_* for each target, representing its proximity to the ideal solution:

(33) $$C_{i}=d_{i}^{-}/\left(d_{i}^{+}+d_{i}^{-}\right)$$

#### 3.2.2. Target Threat Level Assessment Based on D-S Evidence Theory

D-S evidence theory was proposed and improved by Dempster and Shafer. It can handle incomplete, uncertain, and unclear information in multisource evidence, reducing the interference of conflicting weights in decision-making. This enables the fusion of multisource information.

D-S evidence theory first defines a finite non-empty set of hypotheses as the Frame of Discernment (FoD). This set contains *U* mutually exclusive and exhaustive hypotheses.

(34) $$\Theta =\left\{H_{1},\;H_{2},\;\ldots ,\;H_{U}\right\}$$

where *U* is the number of hypotheses in the system, and *H_i_* (*i* = 1, 2, …, *M*) represents the *i*-th hypothesis reflecting the *i*-th possible identification result. According to the definition of FoD, the power set can be denoted by 2^ϴ^.

(35) $$2^{\Theta }=\left\{\Phi ,H_{1},H_{2},\ldots ,H_{U},\left\{H_{1},H_{2}\right\},\left\{H_{1},H_{3}\right\},\ldots ,\left\{H_{1},H_{2},\ldots ,H_{U}\right\}\right\}$$

where Φ is the empty set, and *H* ⊆ ϴ, *H* ∈ 2ϴ.

To describe the support for the hypotheses, a basic probability assignment (BPA), also known as a basic belief assignment (BBA), is introduced into the power set 2^Θ^. The mass function *m*: 2^Θ^→[0, 1] must satisfy the following conditions:

(36) $$m\left(\Phi \right)=0$$

(37) $$\sum_{H\subseteq \Theta }m\left(H\right)=1$$

where *H* is a proposition in 2^Θ^ containing one or more hypotheses, and *m*(H) denotes the initial support for *H*.

Equations (36) and (37) reflect the non-negativity and normalization of the mass function. Without further information, *m*(H) cannot be further subdivided into any proper subsets of *H*. When *m*(*H*) > 0, *H* is called a focal set, and all focal sets are collectively referred to as the core of the mass function.

The belief function *Bel* and plausibility function *Pl* in the power set 2^Θ^ are defined by the following formulas:

(38) $$Bel\left(H\right)=\sum_{A\subseteq H}m\left(A\right)$$

(39) $$Pl\left(H\right)=\sum_{A\cap H\neq \Phi }m\left(A\right)$$

where *H* and *A* are both propositions in the power set 2^Θ^.

**Figure 4:**
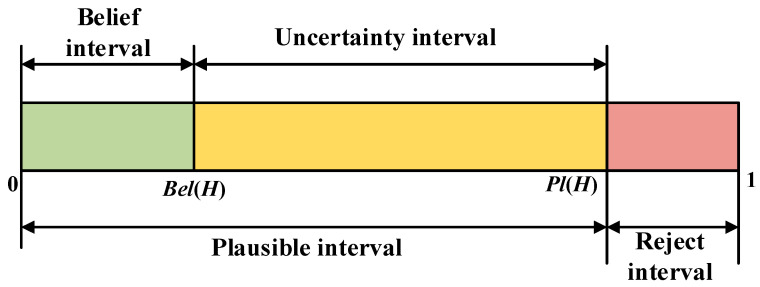
Relationship between *Bel*(*H*) and *Pl*(*H*).

From Equation (38), *Bel*(H) is defined as the degree of belief in proposition *H*, which is the sum of basic probabilities of all subsets of *H*, representing the total belief in *H*. Therefore, it can also be considered the lower-bound function of *H*. *Pl*(*H*) is defined as the plausibility function of *H*, representing the degree of non-false belief in proposition *H*; thus, it is also known as the upper-bound function of *H*. The relationship between *Bel*(*H*) and *Pl*(*H*) can be understood from Figure 4.

The uncertainty description reveals that D-S evidence theory can reflect probabilistic uncertainty, so it can be seen as an uncertainty reasoning theory, showing its applicability to multisource data information fusion. As Figure 4 shows, the logical relationship between *Bel*(*H*) and *Pl*(*H*) is as follows:

(40) $$Bel\left(H\right)\leq Pl\left(H\right)$$

(41) $$Pl\left(H\right)=1-Bel\left(\bar{H}\right)$$

where $\bar{H}$ is the complement of *H*.

The interval [*Bel*(*H*), *Pl*(*H*)] is called the confidence interval or uncertainty interval, representing the uncertainty and imprecision in D-S evidence theory. Suppose that *m*_1_, *m*_2_, …, *m_N_* are *N* independent BPAs obtained by *N* different sensors for the same target. D-S evidence theory’s combination rule (orthogonal sum rule) can be expressed as

(42) $$m=m_{1}⊕m_{2}⊕\ldots ⊕m_{N}$$

Thus, the combination rule for different *m_i_*, *m_j_* (*I*, *j* = 1, 2, …, *N*) can be defined as

(43) $$m_{ij}\left(H\right)=\frac{1}{1-k}\sum_{H_{i}\cap H_{j}=A}m_{i}\left(H_{i}\right)\cdot m_{j}\left(H_{j}\right)\begin{array}{cc} & A\subseteq \Theta ,A\neq \Phi \end{array}$$

(44) $$m\left(\Phi \right)=0$$

where *k* is the total conflict factor, which represents the total conflict between *m_i_* and *m_j_*.

(45) $$k=\sum_{H_{i}\cap H_{j}=\Phi }m_{i}\left(H_{i}\right)\cdot m_{j}\left(H_{j}\right)$$

The value of *k* represents the degree of conflict between two pieces of evidence, with 1/(1 − *k*) serving as the normalization factor. This ensures that the combined evidence *m* in Equation (45) is non-negative and normalized.

D-S evidence theory’s combination rule satisfies commutative and associative laws during computation, defined as, respectively,

(46) $$\left(m_{1}⊕m_{2}\right)⊕m_{3}=m_{1}⊕\left(m_{2}⊕m_{3}\right)$$

(47) $$m_{1}⊕m_{2}=m_{2}⊕m_{1}$$

## 4. Results and Discussion

The purpose of this section is to verify the effectiveness of multi-dimensional feature fusion and the performance of the proposed algorithm through simulation calculations based on existing data and the analysis of measured data. Firstly, the quantitative values of LSS target attributes and flight scenario parameters in the existing data are introduced. Based on the aforementioned subjective and objective weighting methods and the combined weighting method, the index weights of LSS targets are established. Subsequently, the TOPSIS method and D-S evidence theory are used to complete the threat assessment of LSS targets, and comparisons are made with various threat assessment algorithms to verify the feasibility of the fusion method proposed in this paper. On this basis, the measured data are used to calculate the target threat level, and the performance of the proposed algorithm is analyzed and compared with that of other algorithms, the results of which verify the superiority of the method in this paper.

### 4.1. Simulation Example

#### 4.1.1. Data Preprocessing

A set of LSS target detection images were collected in an air defense area, and their original situational data were obtained by integrating various sensor inputs. Using the collected LSS target data, case simulations were conducted to evaluate and rank target threat levels using the proposed method. Table 5 presents the sampled LSS target situational data.

Membership functions for the threat attributes of each target indicator were quantified using the calculation methods described in Section 2.2. Thus, the attribute values in Table 5 were converted into a target attribute decision matrix, listed in Table 6.

#### 4.1.2. Combined Weighting Results

Using the improved AHP method described in Section 3.1.1 and integrating expert opinions, the seven indicators are ranked by importance for target threat assessment in descending order: target distance, target type, velocity component, target altitude, maneuverability, query performance, and protected object. Their relative importance scores are shown in Table 7.

Thus, the weight vector for each indicator can be derived. Table 8 presents the weight calculation results for each indicator using the improved AHP method, with the weight values denoted as *W*_1_:

(48) $$W_{1}={\left[0.4587\;0.1529\;0.1147\;0.0917\;0.0655\;0.0655\;0.0509\right]}^{T}$$

After normalizing the attribute matrix, the weight vectors of each index of the entropy weight method are obtained. Table 9 summarizes the weight calculation results from the entropy weight method, with the calculated weights denoted as *W*_2_:

(49) $$W_{2}={\left[0.1377\;0.0827\;0.3516\;0.2028\;0.1139\;0.1009\;0.0104\right]}^{T}$$

CRITIC calculates the weight vector of each index. Table 10 summarizes the weight calculation results from the CRITIC method, with the calculated weights denoted as *W*_3_:

(50) $$W_{3}={\left[0.184\;0.1729\;0.1264\;0.1909\;0.135\;0.1154\;0.0754\right]}^{T}$$

The combined weight vector *v* of each index is obtained by substituting the above weighting calculation results of the improved AHP, entropy, and CRITIC methods into Equation (29):

(51) $$v=\left[0.259\;0.136\;0.197\;0.162\;0.105\;0.094\;0.046\right]$$

#### 4.1.3. Analysis of Threat Assessment Results

The TOPSIS method ranks the threat levels of eight LSS targets based on the criterion that a closer proximity to the positive ideal solution corresponds to a larger closeness coefficient and a higher threat level. Threat levels are then ordered sequentially based on this coefficient. Table 11 lists the positive/negative ideal solutions for the seven evaluation indicators, and Table 12 presents the threat level calculation results and rankings for the eight LSS targets.

**Figure 5:**
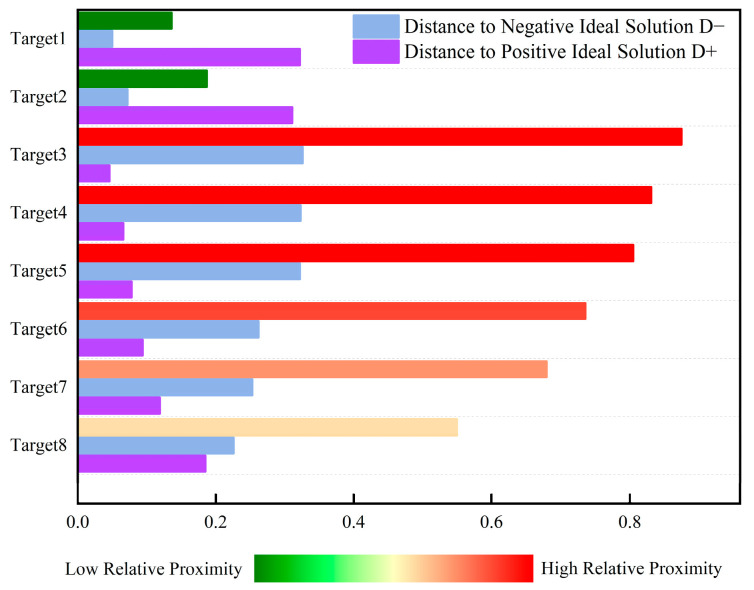
Histogram of target threat level ranking based on TOPSIS evaluation.

Figure 5 shows the numerical comparison of positive and negative ideal solutions and the relative closeness degrees of the seven evaluation indicators for the eight LSS targets. This ranking histogram displays the distribution of multi-target threat levels for intuitive understanding. As an auxiliary tool for dynamic perception, it can effectively help decision-makers make accurate judgments and decisions based on real-time state changes. Target 3 has a closeness coefficient of 0.875; target 4, 0.831; target 5, 0.805; target 6, 0.736; target 7, 0.680; target 8, 0.550; target 2, 0.187; and target 1, 0.136. Thus, the threat level ranking of airborne LSS targets is target 3 > target 4 > target 5 > target 6 > target 7 > target 8 > target 2 > target 1.

The FoD is defined as the set of all threat assessment indicators, i.e., Θ = {target distance, target type, velocity component, target altitude, maneuverability, query performance, protected object}. The results from the improved AHP, entropy weight, and CRITIC weighting methods are treated as BPA functions in D-S evidence theory, such that *m*_1_(*H_j_*) = *W*_1_(*H_j_*), *m*_2_(*H_j_*) = *W*_2_(*H_j_*), *m*_3_(*H_j_*) = *W*_3_(*H_j_*). Pairwise fusion evidence is then calculated using Equations (43)–(48). The weighted conversion matrix *v*_DS_ is then obtained:

(52) $$v_{DS}=\left(0.256\;0.048\;0.112\;0.077\;0.022\;0.018\;0.001\right)$$

**Figure 6:**
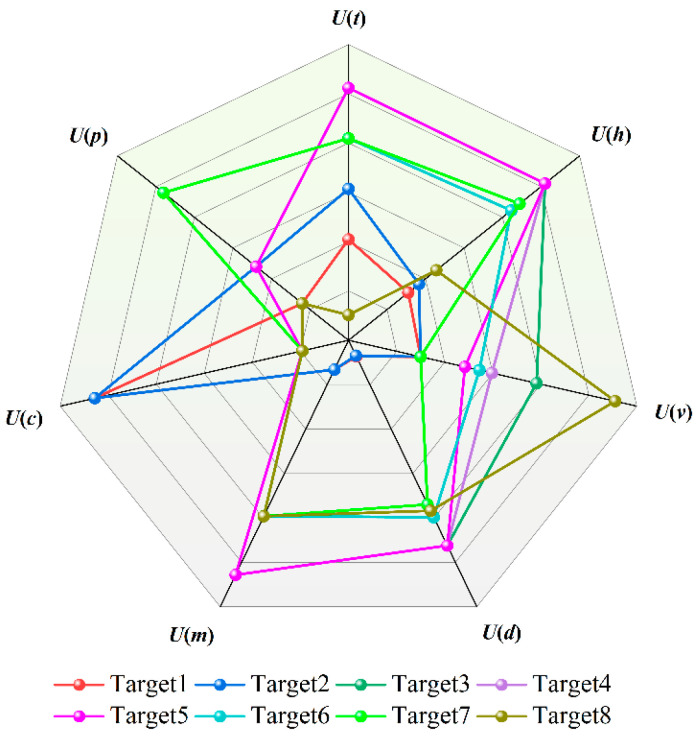
LSS Target threat assessment radar chart.

**Figure 7:**
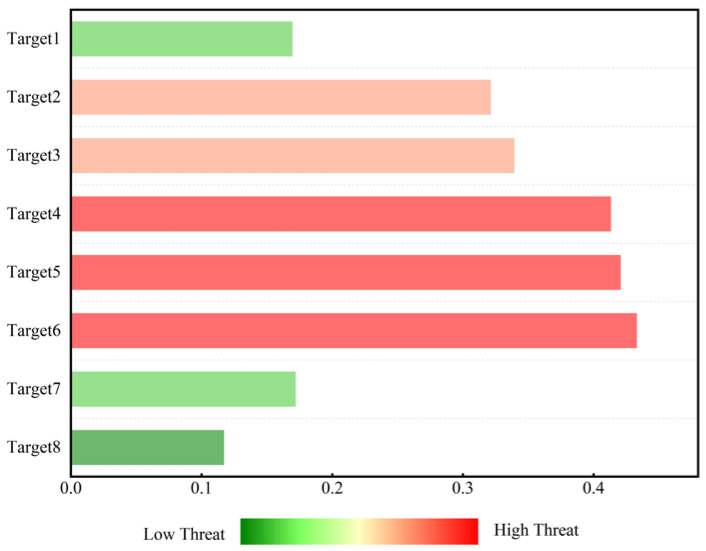
Histogram of threat level ranking evaluated by D-S evidence theory.

Table 13 lists the normalized values of the threat assessment indicators for LSS targets and the calculated threat levels (weighted sums). Displaying the evaluation indicators of LSS targets in the form of a radar chart (as shown in Figure 6) allows for an intuitive observation of the high-dimensional information of target threats. It can be intuitively seen from Figure 6 that when evaluating target threat levels, the deviation in results is mainly reflected in objective factors, such as target altitude and target speed. The histogram of LSS target threat level ranking can be dynamically updated, driven by real-time data. The changing trend in target threat levels can be easily displayed through the threat value scale and color temperature. It can be intuitively seen from Figure 7 that the threat values for Targets 4, 5, and 6 place them in a dangerous state, while Target 8 is in a safe state. The ranking of LSS targets by threat level is Target 3 > Target 4 > Target 5 > Target 6 > Target 7 > Target 2 > Target 8 > Target 1. Targets 3, 4, and 5—all hexacopter UAVs—are classified as high-threat targets due to their low altitude, short distance, and high speed.

A comparison of the threat assessment sequences derived from D-S evidence theory and the TOPSIS method shows that the main difference between the two approaches lies in the assessment of Target 2 (airship) and Target 8 (unmanned aerial vehicle), with their ranking positions swapped, while the ranking order of the other targets remains entirely consistent between methods. Specifically, the assessment consistency of the two methods reaches 75%.

**Figure 8:**
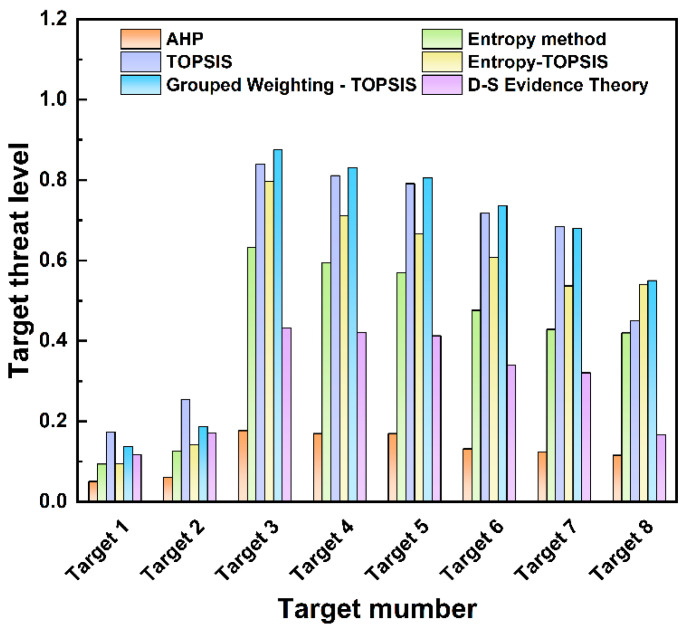
Comparison of threat assessment results of different methods.

The reasons for the discrepancy are as follows: Based on combined weights, the TOPSIS method amplifies the contribution of dominant indicators (for example, the weight of target distance accounts for 24.6%), leading to the threat values of high-threat targets being significantly higher than those of other targets. D-S evidence theory, on the other hand, regards the three types of weights as independent evidence sources and dynamically adjusts the weights through conflict coefficients and orthogonal sum rules. The normalization factor weakens the impact of conflicting evidence, resulting in more balanced outcomes. Secondly, in the TOPSIS method, the inherent characteristics of the target have a substantial influence on the evaluation of the threat value; for instance, the weight of the target type reaches 24.6%, which significantly affects the final calculation of the threat value. D-S evidence theory reduces the dominance of a single indicator through fusion and increases the proportion of dynamic indicators (such as speed and distance). Finally, the TOPSIS method relies on preset weights and has low fault tolerance for data uncertainty. D-S evidence theory quantifies uncertainty through the Bel(H) and Pl(H) intervals, thereby reducing the impact of extreme values. The threat assessment results of the proposed TOPSIS method based on combined weighting and D-S evidence theory are compared with those of the traditional TOPSIS, entropy-weighted TOPSIS, entropy weight, and AHP methods. The results of the comparison are shown in Figure 8, with detailed threat level data listed in Table 14.

Analysis of Figure 8 and Table 14 reveals that when measuring threat levels using the AHP method, different targets have relatively close threat levels—for example, Targets 4 and 5 both have a threat level of 0.170. The entropy weight method shows a significant gap between low- and high-threat targets, though differences among targets with the same threat level are less pronounced (e.g., Target 1 has an extremely low threat level of 0.094). The simulation results indicate consistent outcomes across methods. Notably, the results from D-S evidence theory are more neutral, effectively integrating reasonable components from each weighting method while avoiding large deviations caused by uncertainties in individual approaches. This illustrates that both proposed methods can accurately identify the threat level of hexacopter UAVs in the threat assessment of LSS targets, which verifies the model’s ability to capture key threat features and demonstrates its rationality and credibility. The above analysis indicates that the TOPSIS method provides a significant degree of discrimination in threat values, making it suitable for environments where rapid distinction between high- and low-threat targets is required. In contrast, D-S evidence theory has strong anti-interference ability, which makes it applicable to environments where a balance between subjective and objective uncertainties is needed.

#### 4.1.4. Parameter Sensitivity Analysis

To verify the rationality of the membership function parameters, we conducted a comprehensive sensitivity analysis to evaluate the robustness of the final threat level ranking when the most critical parameters are changed. The One-Factor-At-a-Time (OFAT) method was used to analyze the simulation data in 4.1. Two objective membership functions (target altitude and target distance) and two subjective membership functions (target type and target maneuverability) from Section 2.2 were selected as the targets for sensitivity analysis. Each parameter was varied based on its defined baseline value (50 m, 100 m, 1.0, and 0.8, respectively), while all other parameters remained unchanged. The entire evaluation workflow was executed for each perturbed value, and the new threat levels were recorded.

The sensitivity verification results were quantitatively analyzed through three methods: (1) the Average Rank Change (ARC) of all targets; (2) Spearman’s rank correlation coefficient (*ρ*) between perturbed and baseline rankings; (3) stability of the top three high-threat targets.

Using the data in Table 6 as the baseline input, 2–5 values were selected for each parameter to be analyzed within its fluctuation range. Subsequently, a complete threat assessment model was run for each parameter value, obtaining the threat level rankings of all eight targets, which were finally compared and analyzed with the results in Table 12. The quantitative results of each parameter’s sensitivity are shown in Table 15.

This analysis indicates that the proposed threat assessment model is highly stable against perturbations of key parameters in objective membership functions. The Average Rank Change (ARC) values are small (ARC < 0.5), and within the test range, Spearman’s rank correlation coefficient ρ remains within the ideal range (ρ > 0.97). The top three most threatening targets remained consistent.

In contrast, the threat value of the hexacopter UAV within the subjective membership functions showed higher sensitivity. However, even with a 10% reduction in threat value, only the rankings of Targets 4 and 5 were changed, without affecting the identification of the target posing the most severe threat. This sensitivity analysis demonstrates that the membership function parameters determined in this study are not arbitrarily selected but possess a certain degree of stability within a reasonable value range. This conclusion lays the foundation for the subsequent validation of the model’s effectiveness and reliability.

### 4.2. Threat Level Assessment and Analysis of Measured Data

#### 4.2.1. Pretreatment of Measured Data

**Figure 9:**
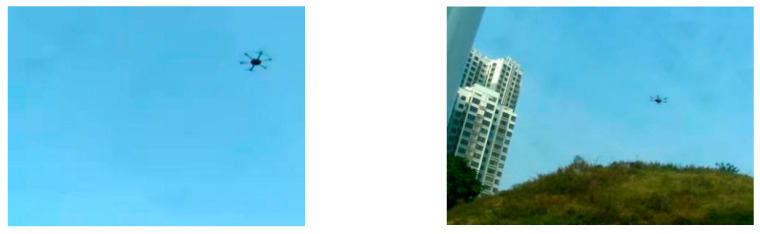
Image acquired by hexarotor UAV.

To validate the effectiveness of the two proposed methods, a threat assessment was conducted on experimentally collected data. All data in this experiment were derived from hexacopter UAV targets, as shown in Figure 9, so all static indicators are identical. Here, no analytical modeling was performed for static indicators of hexacopter UAVs—only dynamic indicators were analyzed and modeled. After target recognition of UAV visible-light images, a model for target dynamic threat indices was developed based on measured experimental data. Table 16 lists the measured target data.

It is acknowledged that the measured data used in this study have certain limitations. The experimental data were collected for only one LSS target type—hexacopter UAVs; thus, the measured data only included dynamic threat indicators (i.e., target distance, altitude, and projected area). Static or discrete attributes such as target type, query capability, and protected objects were not detected in the measured data. This limitation mainly stems from the challenges in acquiring comprehensive, real-time multi-attribute data covering various LSS target types in practical operational environments. Although the measured data failed to effectively demonstrate the validity of static or discrete attributes in threat level assessment, such attributes are mostly subjective factors, whose accuracy mainly relies on the knowledge and experience-based judgments of domain experts. Therefore, the measured data on threat level ranking results for specific LSS targets (hexacopter UAVs), combined with reasonable subjective experience, are sufficient to verify the model’s effectiveness in evaluating high-priority dynamic characteristics.

The attribute values in Table 16 are quantified to obtain the target attribute decision matrix shown in Table 17.

#### 4.2.2. Threat Level Assessment Results

Analysis shows that the importance of the three indicators relative to each other is as shown in Table 18.

Using Equations (17)–(30), the combined weight vector *v* for each indicator is calculated:

(53) $$v=\left[0.506\;0.343\;0.151\right]$$

Based on the obtained combined weight vector *v*, the TOPSIS method is used to calculate and rank the threat levels of the seven hexacopter UAV targets. The results are listed in Table 19 and Table 20.

**Figure 10:**
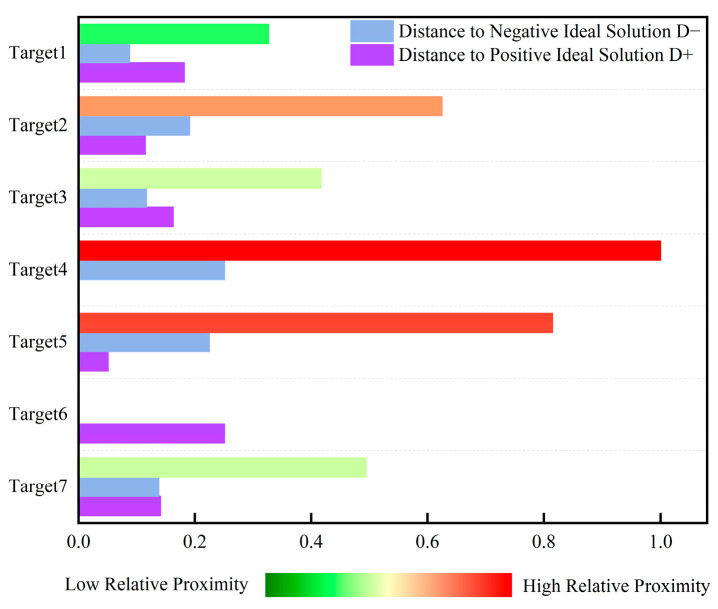
Histogram of TOPSIS evaluation target threat level ranking based on actual measurement data.

It can be seen from Figure 10 that the closeness degree of Target 4 is 1, Target 5 is 0.815, Target 2 is 0.625, Target 7 is 0.495, Target 3 is 0.417, Target 1 is 0.327, and Target 6 is 0.251. Thus, the threat levels of the seven incoming targets are ranked as follows: Target 4 > Target 5 > Target 2 > Target 7 > Target 3 > Target 1 > Target 6. Taking Target 4 as an example, its closeness degree of 1 indicates that it poses the greatest threat among all targets. Analysis of situational data reveals that Target 4 has a minimal vertical altitude and horizontal distance to the visible-light detection platform, and its imaging area in the detectors is the largest. These characteristics justify the maximum threat level for Target 4, consistent with the simulation results.

Using D-S evidence theory, each BPA is first defined as *m*_1_(*H_j_*) = *W*_1_, *m*_2_(*H_j_*) = *W*_2_, and *m*_3_(*H_j_*) = *W*_3_. Then, applying the orthogonal sum rule, we first calculate *k*123 and *m*123:

(54) $$k_{123}=\sum_{H_{j}\neq H_{k}}m_{12}\left(H_{j}\right)\cdot m_{3}\left(H_{k}\right)=0.688$$

(55) $$m_{123}\left(H_{j}\right)=\left(\begin{array}{ccc} 0.151 & 0.343 & 0.506 \end{array}\right)$$

Finally, the threat level calculation results for each target are listed in Table 21.

**Figure 11:**
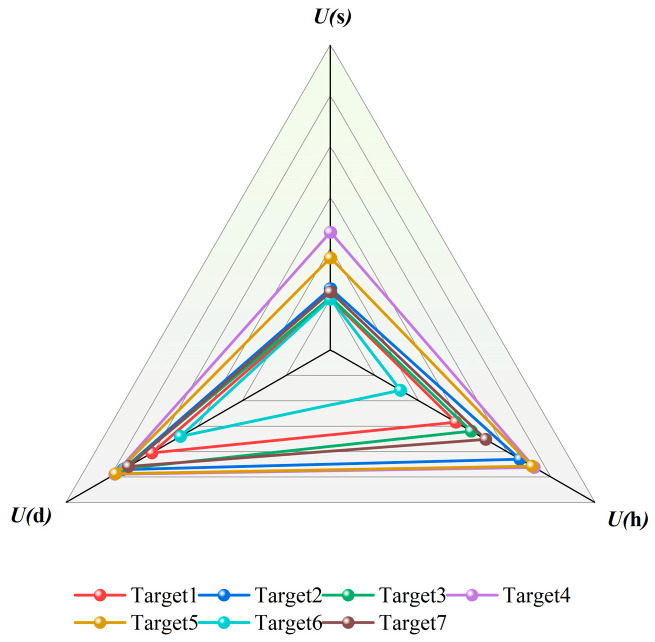
Radar chart of measured target threat assessment.

Based on the attributes and specific values of the measured data, the radar chart for evaluating the threat level of each target is shown in Figure 11. It can be seen from the figure that for hexacopter UAV targets, subjective factors such as target type and maneuverability remain fixed values. Factors affecting the target threat level are largely objective factors, such as target altitude, target distance, and target area. The target area factor is related to target speed and angle and, as shown in the radar chart, varies in a small range for targets of the same type; thus, the factors affecting the target threat level depend on changes in target altitude and target distance.

**Figure 12:**
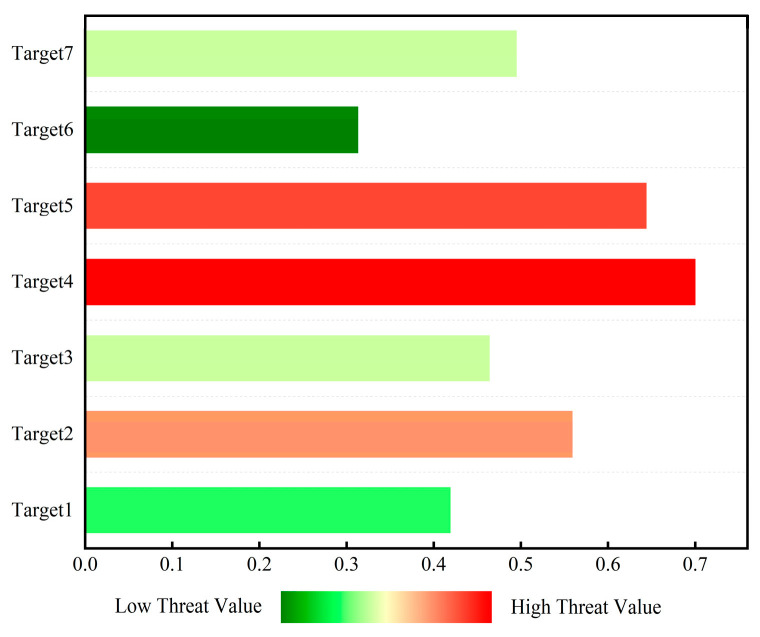
Histogram of threat level ranking evaluated using D-S evidence theory based on measured data.

Figure 12 shows the histogram of threat level ranking evaluated by D-S evidence theory. As shown in the figure, Target 4 has a threat value of 0.7, shown in deep red, indicating the highest threat ranking. Targets 5 and 2 have threat values of 0.644 and 0.559, respectively. The red color indicates that these two targets have a lower threat ranking than Target 4 but are also in a threatening state. However, Target 6 has a threat value of 0.313, shown in deep green, indicating that its threat level is low and it is in a safe state.

#### 4.2.3. Comparative Analysis

**Figure 13:**
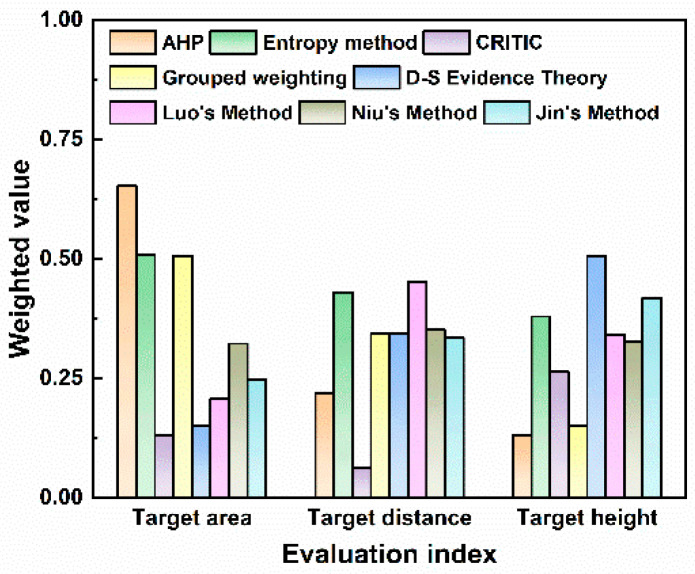
Weight distribution of different methods.

The measured environmental configuration for UAVs is similar to that in many related algorithm studies [29,53]. To further verify the effectiveness of the method proposed in this paper and consider the impacts of the individual AHP, entropy, CRITIC, and TOPSIS methods on the target threat assessment results, the measured data are taken as input, and the calculation and ranking results of the two methods for target threat assessment are compared with those reported in references [29,53]. The weight results obtained by each weighting method are shown in Figure 13.

The quantitative values of weight allocation reflect the different focuses of various algorithms on multiple attributes of the target. Differences in weight allocation can significantly affect the core logic and results of threat assessment. As shown by the comparison of different algorithms, the AHP method assigns the highest weight to the target area (0.63). The target area is a comprehensive reflection of the target type and maneuverability. Its excessive focus on the target area means that the AHP can be easily disturbed by decoy targets in the process of target threat assessment, which ultimately leads to errors in the ranking of multi-target threat levels. The entropy weight method focuses on the weight of the target distance, so the algorithm provides a passive defense response and easily overlooks high-threat target types. The combined weighting method assigns a weight of 0.41 to target distance and 0.39 to target height. This indicates that the combined weighting method allocates weights in a more balanced manner among different evaluation indicators and can account for the requirements of early-warning distance and high-altitude defense. With D-S evidence theory, the highest weight is assigned to target height (0.50), making this algorithm more suitable for actively scanning low-altitude detection areas. Among the three improved algorithms in the literature, Luo’s algorithm determines the subjective and objective threat factor weights of LSS targets based on AHP and information entropy. This method relies on a complete expert knowledge base, and the reliability will decrease with incomplete expert knowledge bases. Niu’s method allocates weights of 0.322, 0.351, and 0.327 to the three evaluation indicators, respectively. The weight allocation with this method is very balanced, which makes the algorithm less sensitive to key factors. Jin’s method focuses on the measurement of dynamic indicators and allocates higher weights to target height and target distance.

**Figure 14:**
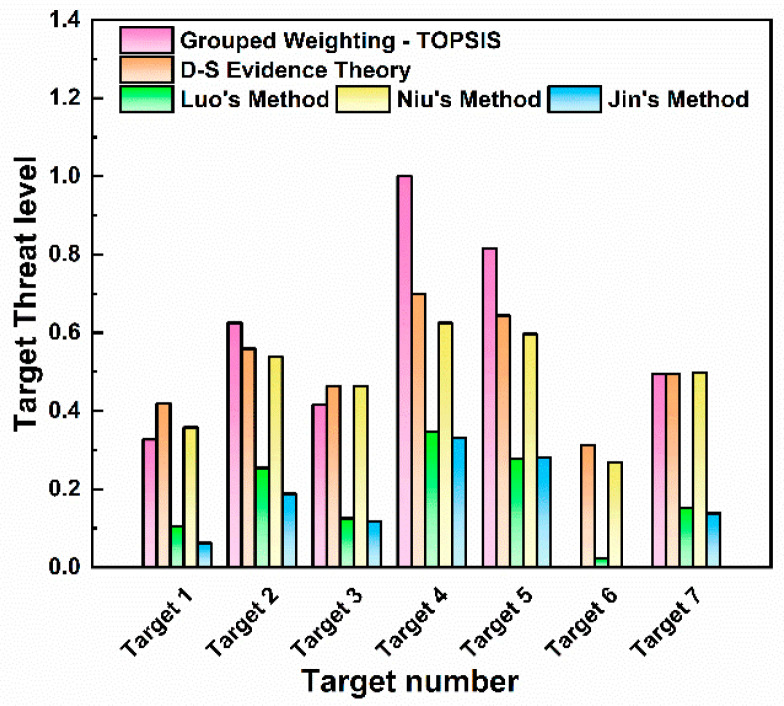
Comparison of threat assessment results for each target.

The threat level calculation and ranking results of the two methods in this paper and the three comparison algorithms are shown in Figure 14. The ranking of threat levels for the seven targets is the same for all algorithms: Target 4 > Target 5 > Target 2 > Target 7 > Target 3 > Target 1. Through the analysis of the histogram, it is found that the TOPSIS threat assessment based on combined weights results in a maximum target threat value of 1 and a minimum of 0. This leads to a large gap in threat values between multiple targets, which can easily cause judgment errors when subject to external interference. In contrast, D-S evidence theory can avoid large deviations caused by irrational factors. The threat values of each target fall within the range of 0.3–0.7, showing a relatively distinct hierarchical distribution. Luo’s method yields relatively low threat values for all targets, with the maximum being 0.346 and the minimum 0.104. This reduces the sensitivity of the algorithm in target threat assessment, as the differences in threat levels between targets are small, so its sensitivity in dynamic target monitoring needs further investigation. Niu’s method is relatively sensitive in responding to UAV threats, but it involves extensive calculation, which may pose computational challenges when dealing with large-scale data. Jin’s method uses the VIKOR model for dynamic attribute threat assessment, and its evaluation results for target motion state information are more reasonable and effective. However, for the discrete measured data in this study, its evaluation results for target threats are relatively low, with the maximum target threat value being only 0.331 and the minimum being 0. Thus, this algorithm is not suitable for the assessment of discrete target threat detection in this study.

### 4.3. Real-Time Performance Verification and Analysis

#### 4.3.1. Experimental Setup

The interception of LSS targets requires the threat assessment system to have extremely high real-time performance. To verify the practical feasibility of the proposed fusion evaluation framework in actual combat and compare the performance of the two evaluation paths (TOPSIS and D-S evidence theory), this section presents the results of strict empirical tests and theoretical analysis of their computational efficiency.

To simulate the actual deployment environment, all experiments were performed on a portable computing platform equipped with an Intel Core (Santa Clara, CA, USA) i7-11800H @ 2.30 GHz CPU and 16 GB RAM. Core matrix operations were conducted in the Matlab environment. The test data included two parts: the eight simulated targets from Section 4.1 and the seven measured targets from Section 4.2 as benchmark data; to evaluate algorithm scalability, large-scale datasets with target scales of 20, 50, 100, and 200 were generated through simulation. The threat attribute dimension was set to 7 in all datasets. The execution time of the complete evaluation process was measured. The process was divided into two stages: weight calculation and threat assessment. To eliminate errors, each dataset was run 200 times, and the average processing time and standard deviation were recorded.

#### 4.3.2. Analysis of Real-Time Performance Results

**Figure 15:**
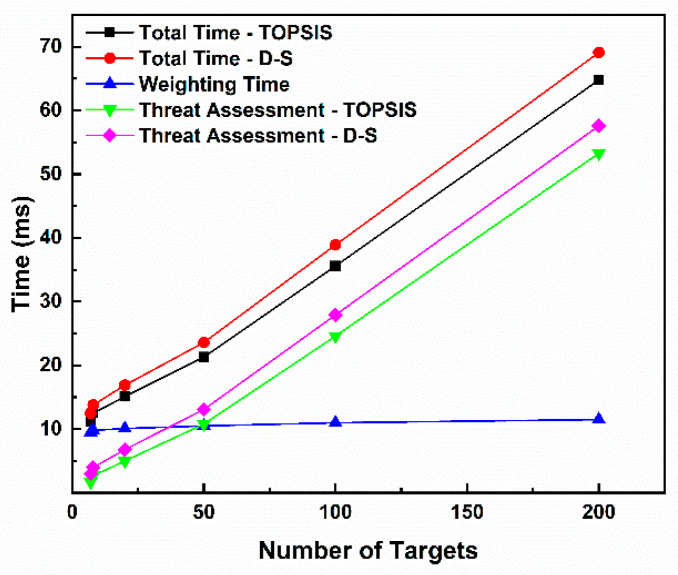
Trend in response time with increasing number of targets.

The measured processing time is shown in Table 22, and its growth trend with the number of targets is shown in Figure 15. Analysis of Figure 15 reveals that both evaluation paths exhibit millisecond-level response speeds. In the benchmark scenario with eight targets, the TOPSIS path takes 12.5 milliseconds, and the D-S path takes 13.8 milliseconds, providing extremely ample response time for the defense system. The total processing time of both paths shows a linear growth relationship with the number of targets (m). Even in large-scale swarming scenarios with up to 200 targets, the processing times of the TOPSIS and D-S paths are only 64.8 milliseconds and 69.1 milliseconds, respectively, which are still far below the typical decision cycle. The TOPSIS path is slightly faster than the D-S path in all test scenarios, with a time difference ranging from approximately 1.3ms to 4.3ms. This is because, after fusing weights, the D-S path needs to calculate the weighted sum for each target, which involves slightly more calculation steps than the distance calculation in TOPSIS. However, this minor performance gap is negligible in actual decision-making scenarios. The time consumed in the weight calculation stage (shared) is less affected by the number of targets, which is consistent with the fact that its complexity mainly depends on the fixed number of indicators (*n* = 7). The time consumed in the threat assessment stage (TOPSIS or D-S) increases linearly with the number of targets, being the main computational load of the algorithm.

A typical air defense command and control system requires the threat assessment process to be completed within 1 to 3 s. Both evaluation paths in the threat assessment framework proposed in this study possess strong real-time processing capabilities, fully meeting the timeliness requirements for rapid and large-scale interception decisions for LSS targets in actual combat environments. Path selection can be based on specific tactical needs rather than performance constraints. The comparative analysis of the TOPSIS method and D-S evidence theory provides practical insights for system deployment. The TOPSIS method, with its significant threat value differentiation capability and power-efficient computation, is particularly suitable for early warning and rapid screening scenarios. It can clearly distinguish between high-threat and low-threat targets, making it well-suited for such time-critical, high-volume screening tasks. In contrast, D-S evidence theory excels in high-risk, uncertain environments. When protecting high-value assets from complex threats that may employ electronic countermeasures, operators benefit not only from threat values but also from their associated uncertainty ranges [Bel(H), Pl(H)]. A target with a moderate threat value but a wide belief–plausibility interval indicates a high degree of information conflict; this inherent ability to quantify and reveal uncertainty makes D-S theory more advantageous in decision-making under ambiguous conditions.

## 5. Conclusions

Based on expert experience, the AHP can be used to subjectively evaluate target attributes. In contrast, the information entropy weight and CRITIC methods can objectively reflect the variability in target attributes, enabling rapid responses to dynamic task planning and maneuverability. Therefore, they are often used for threat estimation when target information is incomplete. This study uses two models—the combined weighting method based on deviation coefficients and D-S evidence theory—to fuse three types of subjective and objective weights, thereby obtaining more reliable evaluation results. The threat levels of LSS targets are calculated using both TOPSIS and D-S evidence theory. Finally, the reliability and effectiveness of the two models are verified through simulation analysis and measured data, providing a reference for LSS target interception decision-making. The main conclusions obtained are as follows:

(1) For the evaluation of LSS target threat levels, the threat level rankings obtained by D-S evidence theory (using basic probability assignments (BPA) and indicator weights) are completely consistent with those of TOPSIS based on the combined weighting method with deviation coefficients. This consistency demonstrates that both methods equally capture core threat features in scenarios without indicator conflicts. The dynamic adjustment of subjective–objective weight ratios via deviation coefficients essentially involves linear weighted summation, while D-S evidence theory achieves evidence fusion through Dempster’s combination rule. Although their mathematical logics differ, in scenarios where all indicators positively support threat levels and no evidence conflict exists, the weighted sum is equivalent to the D-S belief function calculation. Thus, their results align, both providing reliable bases for tactical decision-making.

(2) The combined weighting method based on deviation coefficients calculates the consistency between weighting methods through the dynamic fusion mechanism of the weight deviation similarity index *r_lk_* and allocates combination coefficients according to the degree of correlation, ensuring that the weight allocation between subjective experience and objective data is more in line with actual scenarios. In contrast, D-S evidence theory reduces the influence of contradictory evidence through the conflict coefficient *k*, balances differences between subjective and objective sources, and prevents dominance by a single indicator, aligning with the comprehensive judgment of multisource evidence.

(3) Simulation analysis and comparative experiments show that the two methods proposed in this paper can comprehensively consider the capabilities and intentions of multiple LSS targets and accurately provide threat assessment results based on different LSS movement state information. The proposed methods can be directly applied to real-world low-altitude airspace defense systems.

The limitations of the established expert knowledge base accordingly decrease the reliability of the evaluation. Future in-depth research on the movement laws of LSS targets will continually enrich the expert knowledge base. Future work will focus on three key areas: (1) continuously enrich and improve the expert database and incorporate new threat assessment factors; (2) establish the structure of new threat assessment factor quantification functions and optimize their parameters to improve accuracy; (3) adopt incremental computing technology to accelerate the processing of large-scale data and improve the dynamic update speed of the evaluation system.

## Figures and Tables

**Table 1:** Evaluation of target threat assessment methods.

Theoretical Methods	Advantages	Shortcomings
Evidence Theory [46]	Effectively fuses multisource data and resolves data uncertainty to some extent.	Relies heavily on subjective data.
Fuzzy Reasoning Theory [47]	Can represent qualitative descriptions in the form of quantitative data.	Involves rather subjective definitions of membership functions and fuzzy rules.
Bayesian Network Theory [48]	Derives causal relationships among indicators based on conditional probability theory.	Relies on extensive prior knowledge and data support and has real-time performance issues.
Neural Network [30]	Possesses self-learning and self-adaptive capabilities and can automatically capture complex patterns and correlations through data training.	Has high requirements for the quantity of labeled data; difficult to obtain battlefield environment data.
Multi-Attribute Decision-Making [49]	Suitable for multi-attribute analysis, facilitating direct derivation of threat assessment decisions.	Often has high requirements for data standardization.

**Table 2:** Analysis of LSS target detection capabilities.

Detection Methods	Detection Capabilities
Radar Detection	The reliable detection range is 1–8 km. It can accomplish all-weather rapid detection tasks.
Infrared Detection	The maximum detection range can reach 3.6 km. The detection range is affected by weather conditions, and the recognition capability for small targets is limited.
Visible-Light Detection	The recognition range is usually 100 m–1 km, which is beneficial for target recognition and classification but is susceptible to lighting conditions and weather environments.

**Table 3:** Speed ranges of different LSS targets in low-altitude airspace.

Target Type	Low-Altitude Tethered Balloon	Airship	Low-Altitude Aircraft	Hexacopter Drone	Powered Delta Wing
Speed Range (m/s)	≈1.5 m/s	55 m/s	66 m/s~82 m/s	10 m/s~25 m/s	30 m/s~40 m/s

**Table 4:** Quantitative values for relative importance.

Equally Important	Slightly Important	Important	Strongly Important	Extremely Important	Between Two Degrees of Importance
1	3	5	7	9	2, 4, 6, 8

**Table 5:** LSS target attribute values.

Target	Target Type	High Degree (m)	Azimuthal (°)	Course (°)	Length (m)	Velocity (m/s)	Targeted Mobility	Availability of Target	Protected Object
1	Low-altitude Aerial Photography Balloon	310	125	81	440	25	weak	no	City Center
2	Airship	150	86	113	450	28	weak	no	Major Event
3	Hexacopter UAV 1	42	28	164	45	16	strong	yes	Government Agencies
4	Hexacopter UAV 2	35	146	23	45	13	strong	yes	Government Agencies
5	Hexacopter UAV 3	40	185	78	50	15	strong	yes	Government Agencies
6	Powered Hang Glider 1	68	183	79	140	24	moderate	yes	Field Command Post
7	Powered Hang Glider 2	65	28	90	150	25	moderate	yes	Field Command Post
8	Low-altitude Aircraft	110	71	201	145	30	moderate	yes	Transportation Hub

**Table 6:** Target attribute decision matrix.

Target	*U*(*t)*	*U*(*h*)	*U*(*v*)	*U*(*d*)	*U*(*m*)	*U*(*c*)	*U*(*p*)
1	0.4	0.0146	0	0.0796	0.1	0.3	0.5
2	0.6	0.0909	0	0.0755	0.1	0.3	0.6
3	1	1	0.2877	1	0.8	0.1	0.6
4	1	1	0.1770	1	0.8	0.1	0.6
5	1	1	0.1096	1	0.8	0.1	0.6
6	0.8	0.7553	0.1452	0.8621	0.6	0.1	0.8
7	0.8	0.8163	0	0.8	0.6	0.1	0.8
8	0.1	0.2174	0.4821	0.8316	0.6	0.1	0.5

**Table 7:** Relative importance scores.

Criterion	Target Distance	Target Type	Velocity Component	Target Height	Maneuverability	Query Performance	Protected Object
Target Distance	1	3	4	5	7	7	9
Target Type	1/3	1	4/3	5/3	7/3	7/3	9/3
Velocity Component	1/4	3/4	1	5/4	7/4	7/4	9/4
Target Height	1/5	3/5	4/5	1	7/5	7/5	9/5
Maneuverability	1/7	3/7	4/7	5/7	1	1	9/7
Query Performance	1/7	3/7	4/7	5/7	1	1	9/7
Protected Object	1/9	3/9	4/9	5/9	7/9	7/9	1

**Table 8:** Summary of calculation results of improved AHP method.

Criterion	Eigenvector	Weight Value	Maximum Eigenvalue	CI Value
Target Distance	3.211	45.868%	7	0
Target Type	1.070	15.289%		
Velocity Component	0.803	11.467%		
Target Height	0.642	9.174%		
Maneuverability	0.459	6.553%		
Query Performance	0.459	6.553%		
Protected Object	0.357	5.096%		

**Table 9:** Summary of calculation results of weight calculation by entropy method.

Criterion	Information Entropy *e*	Information Utility Value *d*	Weight Coefficient *w*
Target Distance	0.9055	0.0945	0.1377
Target Type	0.9433	0.0567	0.0827
Velocity Component	0.7589	0.2411	0.3516
Target Height	0.8609	0.1391	0.2028
Maneuverability	0.9219	0.0781	0.1139
Query Performance	0.9308	0.0692	0.1009
Protected Object	0.9929	0.0071	0.0104

**Table 10:** Summary of CRITIC weight calculation results.

Criterion	Index Variability	Index Conflict	Information Volume	Weight
Target Distance	0.396	3.758	1.488	0.184
Target Type	0.327	4.275	1.398	0.1729
Velocity Component	0.169	6.061	1.022	0.1264
Target Height	0.431	3.585	1.544	0.1909
Maneuverability	0.293	3.728	1.091	0.135
Query Performance	0.093	10.077	0.933	0.1154
Protected Object	0.116	5.235	0.610	0.0754

**Table 11:** Positive and negative ideal solutions for evaluation indicators.

Indicator	Positive Ideal Solution A+	Negative Ideal Solution A−
Target Distance	0.259	0.020
Target Type	0.136	0.014
Velocity Component	0.095	0.000
Target Height	0.162	0.002
Maneuverability	0.084	0.011
Query Performance	0.028	0.009
Protected Object	0.046	0.0023

**Table 12:** Calculation results and ranking of target threat levels.

Target	Distance to Positive Ideal Solution d+	Distance to Negative Ideal Solution d−	Relative Proximity	Ranking Structure
Target 1	0.322	0.050	0.136	8
Target 2	0.311	0.072	0.187	7
Target 3	0.046	0.326	0.875	1
Target 4	0.066	0.323	0.831	2
Target 5	0.078	0.322	0.805	3
Target 6	0.094	0.262	0.736	4
Target 7	0.119	0.253	0.680	5
Target 8	0.185	0.226	0.550	6

**Table 13:** Calculation results of threat degree using D-S evidence theory.

Target	*U*(*t*)	*U*(*h*)	*U*(*v*)	*U*(*d*)	*U*(*m*)	*U*(*c*)	*U*(*p*)	Threat Degree (Weighted Sum)	Ranking Structure
1	0.1024	0.0007	0	0.0061	0.0022	0.0054	0.0005	0.1173	8
2	0.1536	0.0044	0	0.0058	0.0022	0.0054	0.0006	0.1720	6
3	0.256	0.0480	0.0322	0.0770	0.0176	0.0018	0.0006	0.4332	1
4	0.256	0.0480	0.0198	0.0770	0.0176	0.0018	0.0006	0.4208	2
5	0.256	0.0480	0.0123	0.0770	0.0176	0.0018	0.0006	0.4133	3
6	0.2048	0.0363	0.0163	0.0664	0.0132	0.0018	0.0008	0.3395	4
7	0.2048	0.0392	0	0.0616	0.0132	0.0018	0.0008	0.3214	5
8	0.0256	0.0104	0.0540	0.0640	0.0132	0.0018	0.0005	0.1696	7

**Table 14:** Threat degrees calculated by different methods.

Method	Target 1	Target 2	Target 3	Target 4	Target 5	Target 6	Target 7	Target 8
AHP Method	0.051	0.061	0.177	0.170	0.170	0.132	0.124	0.116
Entropy Method	0.094	0.126	0.633	0.594	0.570	0.476	0.429	0.420
Single TOPSIS Method	0.173	0.255	0.839	0.811	0.791	0.718	0.684	0.451
Entropy-weighted TOPSIS Method	0.095	0.142	0.797	0.711	0.666	0.608	0.537	0.540
Combined Weighting–TOPSIS Method	0.138	0.187	0.875	0.831	0.805	0.736	0.680	0.550
D-S Evidence Theory	0.117	0.172	0.433	0.421	0.413	0.340	0.321	0.167

**Table 15:** Sensitivity analysis of membership function parameters.

Membership Function	Parameter Value	Average Rank Change	Spearman’s Rank Correlation Coefficient *ρ*	Top 3 Ranking
Baseline	-	0	1.00	T3 > T4 > T5
Target Distance	40 m	0.38	0.98	T3 > T4 > T5
	45 m	0.25	0.99	T3 > T4 > T5
	55 m	0.13	1.00	T3 > T4 > T5
	60 m	0.50	0.97	T3 > T4 > T5
Target Distance	80 m	0.25	0.99	T3 > T4 > T5
	90 m	0.13	1.00	T3 > T4 > T5
	110 m	0.13	1.00	T3 > T4 > T5
	120 m	0.25	0.99	T3 > T4 > T5
Threat Value of Hexacopter UAV	0.9	0.75	0.94	T3 > T5 > T4
	1.1	0.5	0.97	T3 > T4 > T5
Target Maneuverability	0.7	0.46	0.96	T3 > T4 > T5
	0.9	0.32	0.98	T3 > T4 > T5

**Table 16:** Attribute situation values of hexarotor UAVs.

Target	Vertical Height H/m	Horizontal Distance D/m	Target Area S
1	20.3	31.7	416
2	12.9	18.8	939
3	18.5	28.3	540
4	9.7	16.4	1922
5	10.2	16.9	1572
6	26.7	49.7	289
7	14.6	26.8	826

**Table 17:** Experimental target attribute decision matrix.

Target	*U*(*S*)	*U*(*d*)	*U*(*h*)
1	0.201	0.568	0.810
2	0.241	0.859	0.941
3	0.206	0.638	0.933
4	0.463	0.924	0.978
5	0.362	0.914	0.974
6	0.2	0.317	0.680
7	0.228	0.704	0.916

**Table 18:** Relative importance of indicators.

Criterion	Target Area	Target Distance	Target Height
Target Area	1	3	5
Target Distance	1/3	1	5/3
Target Height	1/5	3/5	1

**Table 19:** Positive and negative ideal solutions.

Criterion	Positive Ideal Solution A+	Negative Ideal Solution A−
Area	0.234	0.101
Distance	0.317	0.109
Height	0.148	0.103

**Table 20:** Target threat ranking results.

Target	Distance to Positive Ideal Solution D+	Distance to Negative Ideal Solution D−	Relative Proximity C	Ranking Result
Target 1	0.182	0.088	0.327	6
Target 2	0.115	0.191	0.625	3
Target 3	0.163	0.117	0.417	5
Target 4	0.000	0.251	1.000	1
Target 5	0.051	0.225	0.815	2
Target 6	0.251	0.000	0.000	7
Target 7	0.141	0.138	0.495	4

**Table 21:** Calculation results of threat degree for each target.

Target	*U*(*S*)	*U*(*d*)	*U*(*h*)	Threat Value	Ranking
1	0.201	0.568	0.810	0.419	6
2	0.241	0.859	0.941	0.559	3
3	0.206	0.638	0.933	0.464	5
4	0.463	0.924	0.978	0.700	1
5	0.362	0.914	0.974	0.644	2
6	0.2	0.317	0.680	0.313	7
7	0.228	0.704	0.916	0.495	4

**Table 22:** Data processing test time.

Target Quantity	Total Time—TOPSIS (ms)	Total Time—D-S (ms)	Weight Calculation Time (ms)	Standard Deviation (ms)
7 (Measured)	11.2	13.8	9.5	±0.3
8 (Simulation)	12.5	12.5	9.8	±0.5
20	15.1	16.9	10.1	±0.7
50	21.3	23.6	10.5	±1.2
100	35.6	38.9	11.0	±2.1
200	64.8	69.1	11.5	±3.8

## Data Availability

The original contributions presented in the study are included in the article; further inquiries can be directed to the corresponding author.
